# Novel m7G-related lncRNA signature for predicting overall survival in patients with gastric cancer

**DOI:** 10.1186/s12859-023-05228-w

**Published:** 2023-03-19

**Authors:** Bin Zhao, Fang Fang, Yiqun Liao, Yuji Chen, Fei Wang, Yichao Ma, Chen Wei, Jiahao Zhao, Hao Ji, Daorong Wang, Dong Tang

**Affiliations:** 1 Department of Clinical Medical College, The Yangzhou School of Clinical Medicine, Dalian Medical University, Yangzhou, 225001 China; 2 Department of Clinical Medical College, Yangzhou University, Yangzhou, 225001 China; 3 Department of General Surgery, Northern Jiangsu People’s Hospital Affiliated to Yangzhou University, Yangzhou, 225001 China

**Keywords:** Gastric cancer, Stomach cancer, N7-Methyladenosine, M7G, lncRNA, Biomarker

## Abstract

Presenting with a poor prognosis, gastric cancer (GC) remains one of the leading causes of disease and death worldwide. Long non-coding RNAs (lncRNAs) regulate tumor formation and have been long used to predict tumor prognosis. N7-methylguanosine (m7G) is the most prevalent RNA modification. m7G-lncRNAs regulate GC onset and progression, but their precise mechanism in GC is unclear. The objective of this research was the development of a new m7G-related lncRNA signature as a biomarker for predicting GC survival rate and guiding treatment. The Cancer Genome Atlas database helped extract gene expression data and clinical information for GC. Pearson correlation analysis helped point out m7G-related lncRNAs. Univariate Cox analysis helped in identifying m7G-related lncRNA with predictive capability. The Lasso-Cox method helped point out seven lncRNAs for the purpose of establishing an m7G-related lncRNA prognostic signature (m7G-LPS), followed by the construction of a nomogram. Kaplan–Meier analysis, univariate and multivariate Cox regression analysis, calibration plot of the nomogram model, receiver operating characteristic curve and principal component analysis were utilized for the verification of the risk model’s reliability. Furthermore, q-PCR helped verify the lncRNAs expression of m7G-LPS in-vitro. The study subjects were classified into high and low-risk groups based on the median value of the risk score. Gene enrichment analysis confirmed the constructed m7G-LPS’ correlation with RNA transcription and translation and multiple immune-related pathways. Analysis of the clinicopathological features revealed more progressive features in the high-risk group. CIBERSORT analysis showed the involvement of m7G-LPS in immune cell infiltration. The risk score was correlated with immune checkpoint gene expression, immune cell and immune function score, immune cell infiltration, and chemotherapy drug sensitivity. Therefore, our study shows that m7G-LPS constructed using seven m7G-related lncRNAs can predict the survival time of GC patients and guide chemotherapy and immunotherapy regimens as biomarker.

## Introduction

Gastric cancer (GC) ranks fifth on the list of highly prevalent malignancies and remains the third leading risk factor for cancer-caused fatalities globally [1]. Stomach adenocarcinoma is the main pathologic type of GC originating from the stomach's most superficial glands or mucous membranes. The understanding of the pathogenesis and progression of GC remains limited, due to which a majority of patients are diagnosed with localized or distant metastasis, thus missing the opportunity for radical surgical treatment [2]. Moreover, postoperative recurrence is also a major cause of GC-associated deaths. Although advanced surgical techniques such as hot intraperitoneal chemotherapy, improved systemic chemotherapy, targeted therapy, and immunotherapy have made great strides, the prognosis of GC remains unsatisfactory [3]. Therefore, the identification of novel predictive biomarkers and promising pharmaceutical target agents for GC is extremely important.

RNA methylation is a post-transcriptional modification commonly existing in eukaryotes and prokaryotes [4]. Based on the modification site, RNA methylation can be classified as N6-Methyladenosine (m6A), 5-Methylcytidine (m5C), N7-methylguanosine (m7G), or 2-*O*-Methylation [5]. By directly affecting messenger RNA, ribosomal RNA, microRNA, and transfer RNA metabolism, m7G modification directly functions in several normal physiological mechanisms and pathologies [6]. Multiple research reports have shown the close association of m7G modification with tumorigenesis and cancer growth. Being a prominent mediator of m7G, methyltransferase 1 (METTL1) expression is notably upregulated in hepatocellular carcinoma depicting its relationship with poor prognosis. A study showed that METTL1 suppression in-vitro and in-vivo could effectively limit bladder cancer proliferation, migration, and invasion [7]. In lung cancer, METTL1-mediated m7G promotes miRNA maturation by destabilizing stem-loop structures, thereby inhibiting cell migration and, thus, metastasis [8]. In addition, many bioinformatics investigations have highlighted the use of m7G regulation as a predictive marker for GC, breast cancer, and melanoma gliomas [9, 10].

LncRNAs are greater than 200 nucleotides long and form an important part of the non-coding genome [11]. Typically, lncRNAs regulate the expression of specific miRNAs by acting as competitive endogenous RNAs to target downstream molecules [12]. A lot of research has demonstrated lncRNAs’ function in numerous biological processes such as DNA methylation, histone modification, RNA post-transcriptional regulation, and protein translation regulation, and their involvement in tumorigenesis and progression [13]. Furthermore, RNA methylation of lncRNAs was also found to influence cancer growth. A study has shown that m6A ‘writer’ METTL3 increases LINC00958 stability and promotes hepatocellular carcinoma advancement [14]. LncRNA UBA6-as1 slows down UBA6 mRNA destruction by modifying m6A methylation status, thereby inhibiting the malignancy of ovarian cancer cells [15]. In glioblastoma stem cells, the m6A demethylase ALKBH5 interacts with lncRNA Forkhead box protein M1(FOXM1)-AS for the purpose of promoting cancer cell growth and tumorigenicity [16]. In addition, many bioinformatics analyses have shown that lncRNAs have great potential as prognostic biomarkers for gastric cancer. wang et al. used LASSO analysis to identify four Pyroptosis-Related-lncRNAs (HAND2-AS1, LINC01354, RP11-276H19.1, and PGM5-AS1), and demonstrated that these four lncRNAs could well predict the prognosis of gastric cancer patients, and found that the pyroptosis risk score of gastric cancer was associated with clinicopathological features and TME alterations [17]. And in another study, four ferroptosis-related lncRNAs (AP003392.1, AC245041.2, AP001271.1, and BOLA 3-AS 1) could also well predict the prognosis of gastric cancer patients [18]. Although several studies have identified the role of lncRNA methylation in tumorigenesis and its potential as a biomarker, the role of m7G-related lncRNA in gastric cancer remains unclear.

The tumor immune microenvironment (TIME) includes tumor cells, immune cells, tumor-associated fibroblasts, peripheral microvessels, different cytokines, and extracellular matrix [19]. Tumor-related onset and advancement are influenced by the interactions between its cells and the microenvironment [20]. LncRNA regulates immune cell differentiation, growth, secretory elements, and auxiliary physiological procedures in the TIME, affecting tumor onset and progression [21]. LINC01116 knockdown affects IL-1b release, which promotes the use of tumor-associated neutrophils, which, in turn, results in the accumulation of TAN, causing the production of numerous cytokines, thus leading to tumor growth [22]. The lncRNA HOXA transcript at the distal tip (HOTTIP) enhances IL-6 expression by upregulating PD-L1 expression in neutrophils allowing ovarian cancer cells to escape the immune system. HOTTIP promotes IL-6 secretion, thereby upregulating PD-L1 expression in neutrophils and ultimately promoting the ability of ovarian cancer cells to escape the immune system [23]. In addition, LINC00662 upregulates the expression of WNT3A by attaching to miR-15a, miR-16, and miR-107 competitively, causing stimulation of the Wnt/β-catenin signaling pathway in HCC cells and increased polarization of M2 macrophages, leading to tumor development [24]. However, limited research is available on the association of lncRNAs with immune cell infiltration in GC.

During this research, m7G-related lncRNAs in GC were identified based on The Cancer Genome Atlas (TCGA) database, and a prognostic model containing seven m7G-related lncRNAs was established. The relationship of risk score with immune infiltration, immune checkpoint genes, and chemotherapeutic drug sensitivity was also studied.

## Methodology

### Procurement of data

The data for transcriptome sequencing data and clinical information were taken from the TCGA database (https://portal.gdc.cancer.gov/). Patient clinical information was extracted, including age, gender, stage, grade, TNM stages, and survival status. After downloading, samples with incomplete clinical data, low gene expression, and OS < 30 were excluded. Eventually, RNA sequencing data from 337 GC samples were included. Data on lncRNA annotation was taken from the GENCODE database (https://www.gencodegenes.org).

### Identification of differential expression and interactions of m7G methylation-regulated genes

Based on previous literature, 22 m7G markers were identified. These include AGO2, DCP2, DCPS, EIF3D, EIF4A1, EIF4E, EIF4E2, IF4E3, EIF4G3, GEMIN5, IFIT5, LARP1, METTL1, NCBP1, NCBP2, NCBP3, NUDT10, NUDT11, NUDT16, NUDT3, NUDT4, WDR4. The expression matrix of m7G markers and lncRNAs was obtained from RNAseq. 'Limma' (linear models for microarray data) is a differential expression screening method based on generalized linear model [25]. The R software package ‘limma’ (version 3.40.6) was utilized for differential evaluation for the purpose of obtaining gene expression differences across different comparison groups and control groups. The filtering criteria were set to be |log2fold change|> 1 and false discovery rate (FDR) < 0:05. The STRING database (https://www.string-db.org) helped construct a Protein–Protein Interaction (PPI) network of m7G regulatory genes.

### Acquisition of m7G-LPS and establishment of a predictive risk model

RNAseq data was used to extract the lncRNA expression matrix. Pearson correlation analysis was conducted with the help of the “corrplot” function in R software for obtaining m7G-related lncRNA (r > 0.3, p < 0.05). Survival time, survival status, and gene expression data were integrated with the help of the R software package “survival”, and univariate Cox regression helped in evaluating the predictive efficiency of each gene. The least absolute shrinkage and selection operator (LASSO) Cox regression analysis was conducted with the help of the R package “glmnet.” In addition, tenfold cross-validation was also set for the purpose of developing an optimized model. The Lambda value was set to 0.00281938915464775, after which seven genes were obtained. The formula used for risk score calculation is given below:

$$Risk\,score = \mathop \sum \limits_{i = 1}^{n} Coef_{i} *Exp_{i}$$

where Coefi represents the coefficients, and Expi stands for the FPKM value of each m7G-related lncRNA

### Assessment of the predictive (prognostic) model of m7G-LPS

The receiver operating characteristic (ROC) curve analysis was conducted with the help of the R package “pROC” (version 1.17.0.1) for the purpose of obtaining the area under the curve (AUC). Univariate and multivariate Cox regression analysis confirmed the independent predictive efficiency of the m7G-related lncRNA prediction model for GC. Principal components analysis (PCA) was conducted with the help of the R package “stats” (version 3.6.0). Using the R package “rms”, survival time, survival status, and eight features data were integrated, followed by the establishment of a nomogram using the Cox method for the purpose of assessing the feature’s predictive efficiency in 337 samples.

### Gene set enrichment analysis (GSEA)

The GSEA software (version 3.0) was obtained from the GSEA website (http://software.broadinstitute.org/gsea/index.jsp) [26]. Two groups were established by the division of the samples according to the risk score and the *c5.go.mf.v7.4.symbols.gmt* subset, *c2.cp.kegg.v7.4.symbols.gmt* subset*, c5.go.bp.v7.4.symbols.gmt* subset and *c5.go.cc.v7.4.symbols.gmt* subset was downloaded from the Molecular Signatures Database (http://www.gsea-msigdb.org/gsea/downloads.jsp) for the purpose of evaluating the associated pathways and underlying molecular mechanisms [27–30]. On the basis of gene expression profile and phenotype grouping, the minimum and maximum values of the gene set were set to 5 and 5000, respectively, and 1000 times of resampling was performed. A p value of < 0.05 and an FDR of < 0.25 were considered statistically significant.

### Immunocorrelation analysis and drug sensitivity analysis of prognostic features

The Perl programming language was used for the purpose of obtaining an immune infiltrating cellular matrix and CIBERSORT for immune infiltration analysis. Single-sample GSEA (ssGSEA) helped in assessing the immune cells and their functionality. The “pRRophetic” package helped compare the differences in IC50 values of chemotherapeutic drugs utilized in the treatment of GC. Results of the immunocorrelation analysis were viewed using the R packages “barplot”, “corrplot”, and “ggplot2”.

### qPCR of the expression of m7G-related lncRNAs in tissues

Human normal gastric mucosal epithelial cells GSE-1 and human GC cell lines MKN-45, AGS, and HGC-27 were bought from Shanghai FuHeng BioLogy Ltd. The Trizol reagent (Vazyme Biotech Co., Ltd) was utilized to isolate total cellular RNA. Reverse transcription was conducted following the instructions of the Vazyme reverse transcription kit (Vazyme Biotech Co., Ltd). Quantitative PCR (qPCR) using 2X ChamQ Universal SYBR QPCR Master Mix kit (Analytik Jena AG). The Ct value data in the reaction were collected with a corrected threshold setting, and qPCR was used for relative quantification using the 2^−ΔΔCt^ method. Each PCR reaction was performed in triplicates. For PCR amplification, the primers (“**F**” represents “Forward” and “**R**” represents “Reverse”) used include: *CHROMR* **F** 5′-CTGGTGCTGCTGAGTAACCA-3′ and **R** 5′- AAAGCGAGGACAACCAGAGA -3′, *LINC01094* **F** 5′- GAGGGAGCACTGGGATGTTA -3′ and **R** 5′- CCTTGCAGCTAGGAGTGGAC -3′, *AL355574.1* **F** 5′- GAGTGGAGTTCTTGGGAA -3′ and **R** 5′- GGCCACAGATAACTGCTGCT -3′, *AC245041.1* **F** 5′- GCAAGAGGCAGCTATTGGAC -3′ and **R** 5′- TGTGCAGTGGAGAGATCCTG -3′, *and AL161785.1* **F** 5′- TGATACCTCGCCACATTCTG -3′ and **R** 5′- AAAGCGAGGACAACCAGAGA -3′, *AP001528.1* **F** 5′- CCAGTGGTCCTCCTTTCTGA -3′ and **R** 5′- CATTTCAGCTTGAGGCTTCC -3′, *AC005586.1* **F** 5′- AGCATCGCCAGAGGAAACTA -3′ and **R** 5′- AAGCTTCCAGCTGGCATAAA -3′, and *GAPDH* **F** 5′- CAGCCTCAAGATCATCAGCA -3’ and **R** 5’- TGTGGTCATGAGTCCTTCCA -3′. GAPDH was utilized in the form of internal control to determine relative expression.

### Statistical analysis

Data analysis was done primarily using the R software (version 4.0.3) and Perl software (version 5.3). In this study, univariate and multifactorial Cox regression, Lasso regression, Kaplan–Meier method, PCA, and ROC analysis were used. Kruskal–Wallis test helped compare the differences (variations) across different groups. Pearson correlation test helped in carrying out correlation analysis. The rest of the analyses were performed as described previously. *P* < 0.05 was considered statistically significant (∗ *p* < 0.05, ∗  ∗ *p* < 0.01, and ∗  ∗  ∗ *p* < 0.001).

## Results

### Differential expression and interaction of m7G regulatory genes

**Fig. 1:**
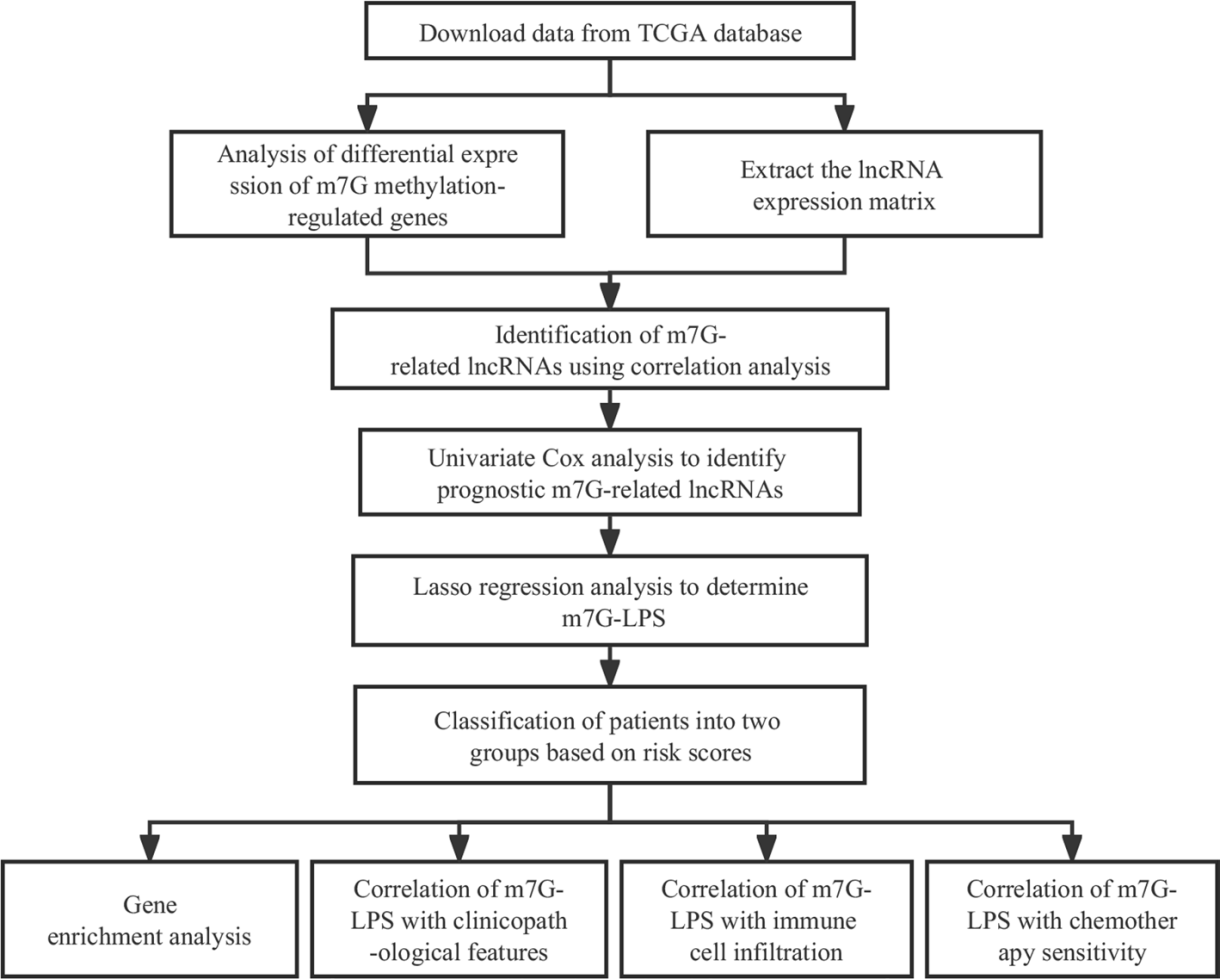
Flow chart

**Fig. 2:**
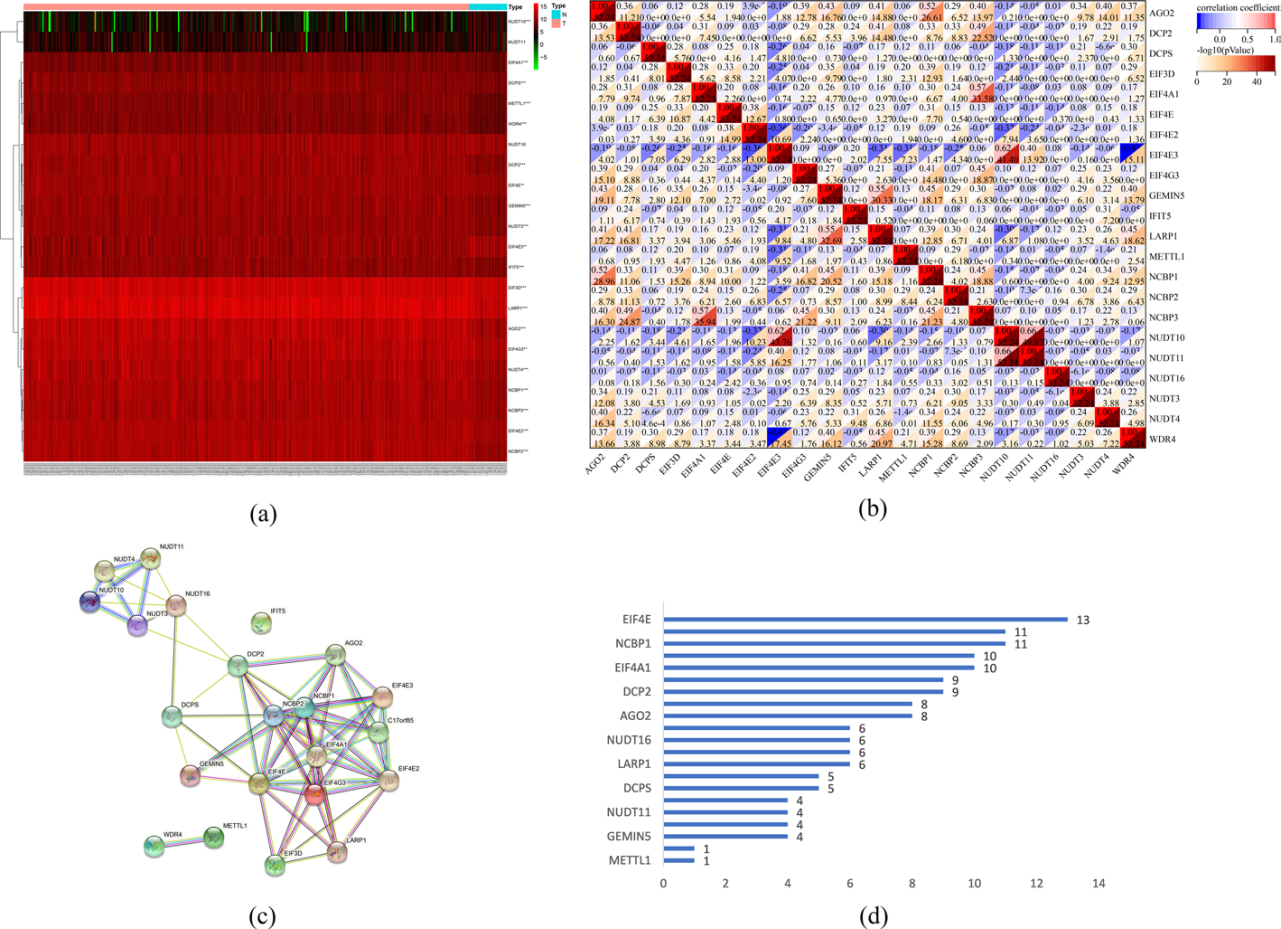
Differential expression and interactions of m7G methylation-regulated genes. **a** Heatmap of differential expression of m7G methylation-regulated genes. **b** Correlation heat map. **c** Protein–protein interaction network (PPI). **d** Node count of PPI network

The flow chart was shown in Fig. 1. Initially, the expression of 22 m7G methylation-regulated genes was analyzed in GC and healthy samples from the TCGA database. A remarkable variation was observed in the m7G regulatory genes between GC and healthy tissues. Particularly, the expression of *AGO2*, *DCP2*, *DCPS*, *EIF3D*, *EIF4A1*, *EIF4E*, *EIF4E2*, *EIF4G3*, *GEMIN5*, *IFIT5*, *LARP1*, *METTL1*, *NCBP1*, *NCBP2*, *NCBP3*, *NUDT3*, *NUDT4*, and *WDR4* was remarkably increased in GC compared to healthy tissues (*p* < 0.001). *EIF4E3* and *NUDT10* expression was significantly decreased in GC compared to healthy tissues. However, no difference in the expression of *NUDT11* and *NUDT16* was found between GC and normal tissues (Fig. 2a). In the correlation analysis of the 22 regulatory genes, *EIF4E* expression was strongly associated with *NUDT10* expression (Fig. 2b). In the next step, the STRING database helped in developing a PPI network to determine the relationship between the identified regulatory genes. A close relationship was found among all regulatory genes except *IFIT5* (Fig. 2c). The node count diagram revealed *EIF4E's* relation to 13 other genes, suggesting that EIF4E may be key in the PPI network (Fig. 2d). It is evident from the above-mentioned findings that m7G methylation-regulated gene expression varied remarkably across GC and healthy tissues, suggesting its involvement in GC onset and advancement.

### Identification of m7G-related lncRNA prognostic signature (m7G-LPS)

**Fig. 3:**
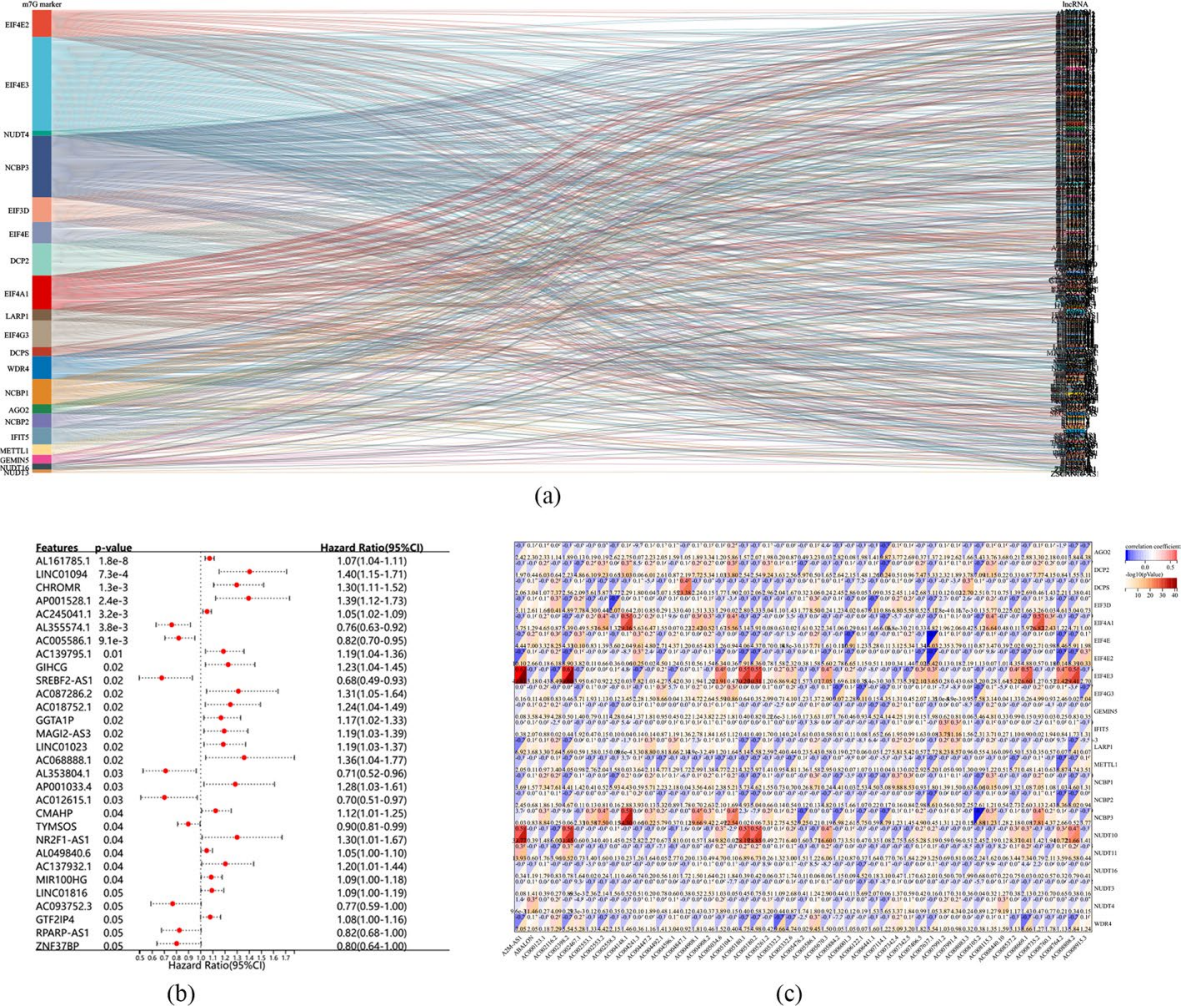
Identification of m7G-related lncRNA. **a** Sankey diagram of m7G gene and m7G-related lncRNA. **b** Univariate COX analysis of prognostic m7G-related lncRNA. **c** Heatmap of correlation between m7G gene and prognostic m7G-related lncRNA

**Fig. 4:**
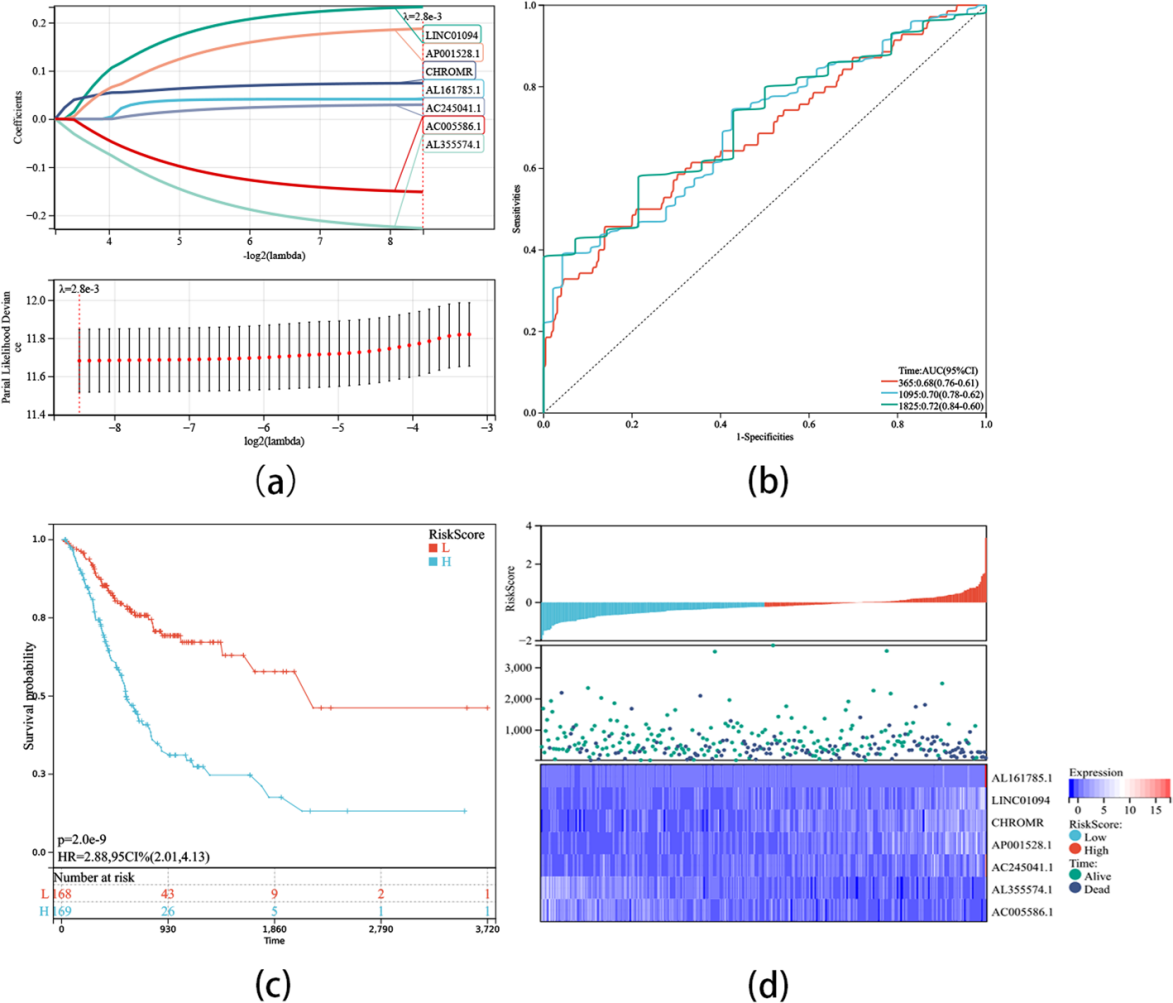
Lasso Regression analysis, Time-dependent ROC analysis, and Kaplan–Meier analysis, risk score analysis for m7G-LPS **a** m7G-LPS lasso regression analysis and Determine the optimal LASSO settings. **b** Time-dependent ROC analysis for m7G-LPS. **c** Kaplan–Meier curves between high and low risk groups **d** Distribution of risk groups, prognosis, and m7G-LPS expression heat maps

Initially, based on the annotation files downloaded from the ‘GENCODE’ website, the lncRNAs expression matrix was identified in the TCGA database, followed by extraction of the expression matrix of 22 m7G regulatory genes from the TCGA database. Those lncRNAs whose expression values were related to one or more m7G methylation-regulated genes were defined as m7G-related lncRNAs (|Pearson R|> 0.3 and p < 0.05). Finally, 446 m7G-associated lncRNAs (Fig. 3a) were identified. Subsequently, univariate COX regression analysis (*p* < 0.05, Fig. 3b) and correlation analysis (Fig. 3c) helped identify 25 lncRNAs having good predictive efficiency. Then, genes with p < 0.01 were screened for Lasso regression analysis, and finally, seven m7G-related lncRNAs, namely, AL161785.1, LINC01094, CHROMR, AP001528.1, AC245041.1, AL355574.1, and AC005586.1, were identified (Fig. 4a). Among these genes, AL355574.1 and AC005586.1 were recognized as protective effects (HR < 1, p < 0:05). In contrast, AL161785.1, LINC01094, CHROMR, AP001528.1, and AC245041.1 were considered as risk effects (hazard ratio, HR > 1, p < 0:05).

The formula used for calculating the GC sample risk score is given below: Riskscore = 0.0410201815783705 × AL161785.1 + 0.232602359496281 × LINC01094 + 0.0744239113701401 × CHROMR + 0.1873048414553 × AP001528.1 + 0.0295140650717325 × AC245041.1–0.226949073687661 × L355574.1–0.151514242977824 × AC005586.1. As per the median value of the risk score, the samples were stratified into two groups, one of high risk and the other of low risk. The Kaplan–Meier survival curve depicted a remarkably shorter overall survival (OS) of the high-risk group in comparison to the other group (p < 0.001, Fig. 4c). As given in the risk value curve and the survival status scatter plot, the survival time and survival status of the high-risk subjects were worse than those in the low-risk category (Fig. 4d). The established prognostic model’s survival prediction capability was evaluated with the help of the ROC curve for GC patients over one, three, and five years, and the AUC values were 0.68, 0.70, and 0.72, respectively (Fig. 4b). These findings indicate that the established m7G-LPS has accurate OS prediction ability.

### Validation of the prediction model constructed using the m7G-LPS and construction of nomograms

**Fig. 5:**
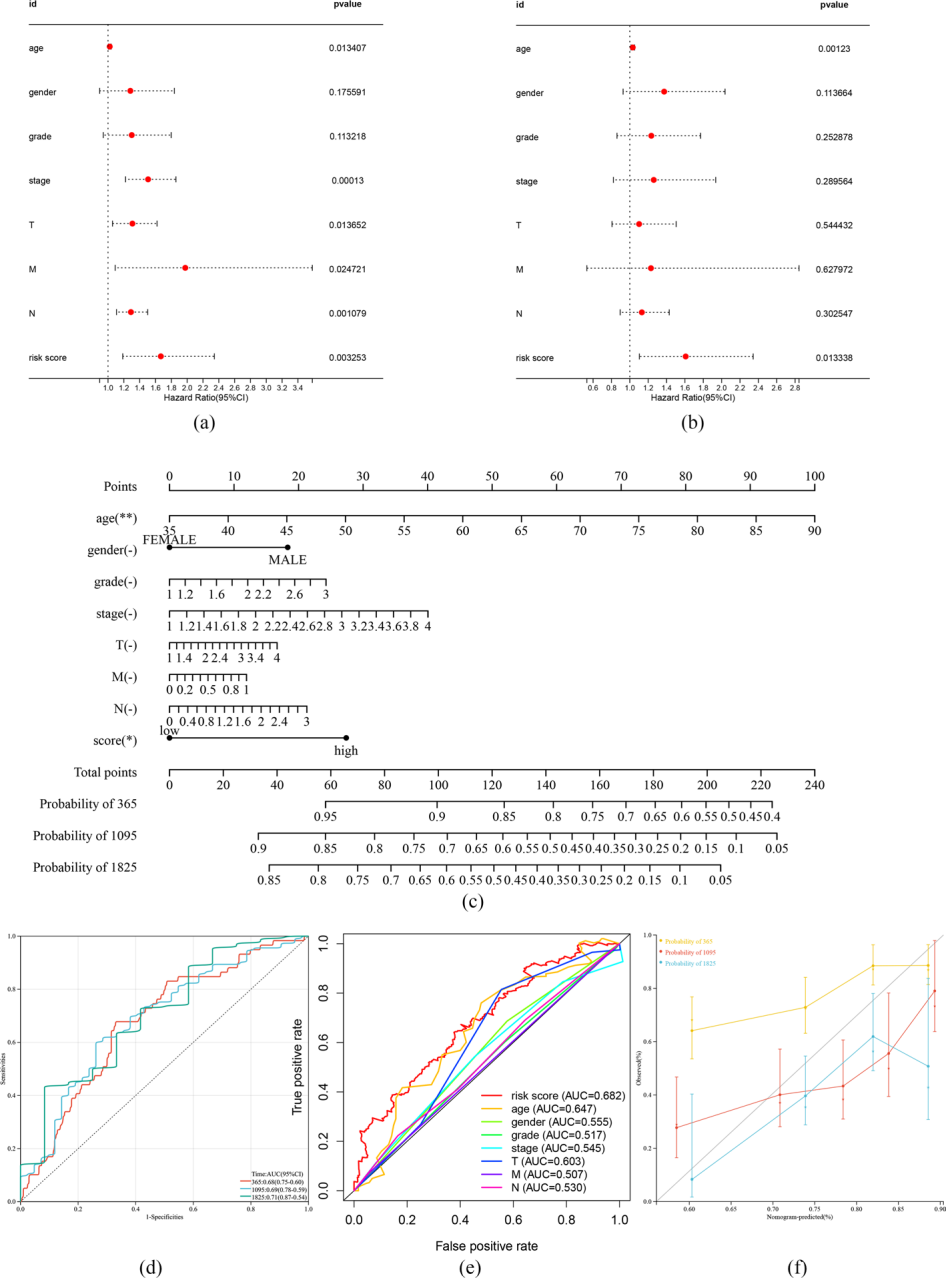
Validation of Prediction Models Constructed with m7G-LPS and Construction of Nomograms. **a** Univariate and **b** multivariate Cox regression analysis between multiple clinical variables and OS in GC patients. **c** Clinical prognostic nomogram predicted the survival risk of GC patients. **d** Time-dependent ROC and **e** Clinical ROC curves are used to evaluate the diagnostic capabilities of models. **f** Calibration plot of the nomogram model. (∗ p < 0.05, ∗  ∗ p < 0.01, and ∗  ∗  ∗ p < 0.001)

To test whether the risk score was an independent risk factor, the survival time, survival status, age, sex, tumor pathological stage, TNM stage, tumor grade, and risk score were integrated and analyzed using univariate and multivariate COX regression. The findings of these analyses (*p* < 0.05) revealed HR = 1.667546, 95% CI 1.186220734–2.344176354 and HR = 0.621721461, 95% CI 0.426695209–0.905886843, respectively, for risk score, from which it can be concluded that risk score can serve as an independent risk factor for GC (Fig. 5a, b). A nomogram was also constructed based on the findings of the Cox regression analysis (Fig. 5c). For the purpose of assessing the prognostic efficiency of the constructed model, the AUC values of the time-dependent ROC curve of the risk score were evaluated, and the values over one, three, and five years were 0.68, 0.69, and 0.71, respectively. Moreover, the risk score AUC in the clinical ROC appeared to be remarkably increased compared to other clinical indicators (Fig. 5d, e). The calibration curve of the nomogram is shown in Fig. 4f. These findings are indicative of the m7G-LPS-based prediction model’s enhanced sensitivity as well as specificity in predicting the prognosis of patients with GC.

### Principal component analysis (PCA) of m7G-LPS

**Fig. 6:**
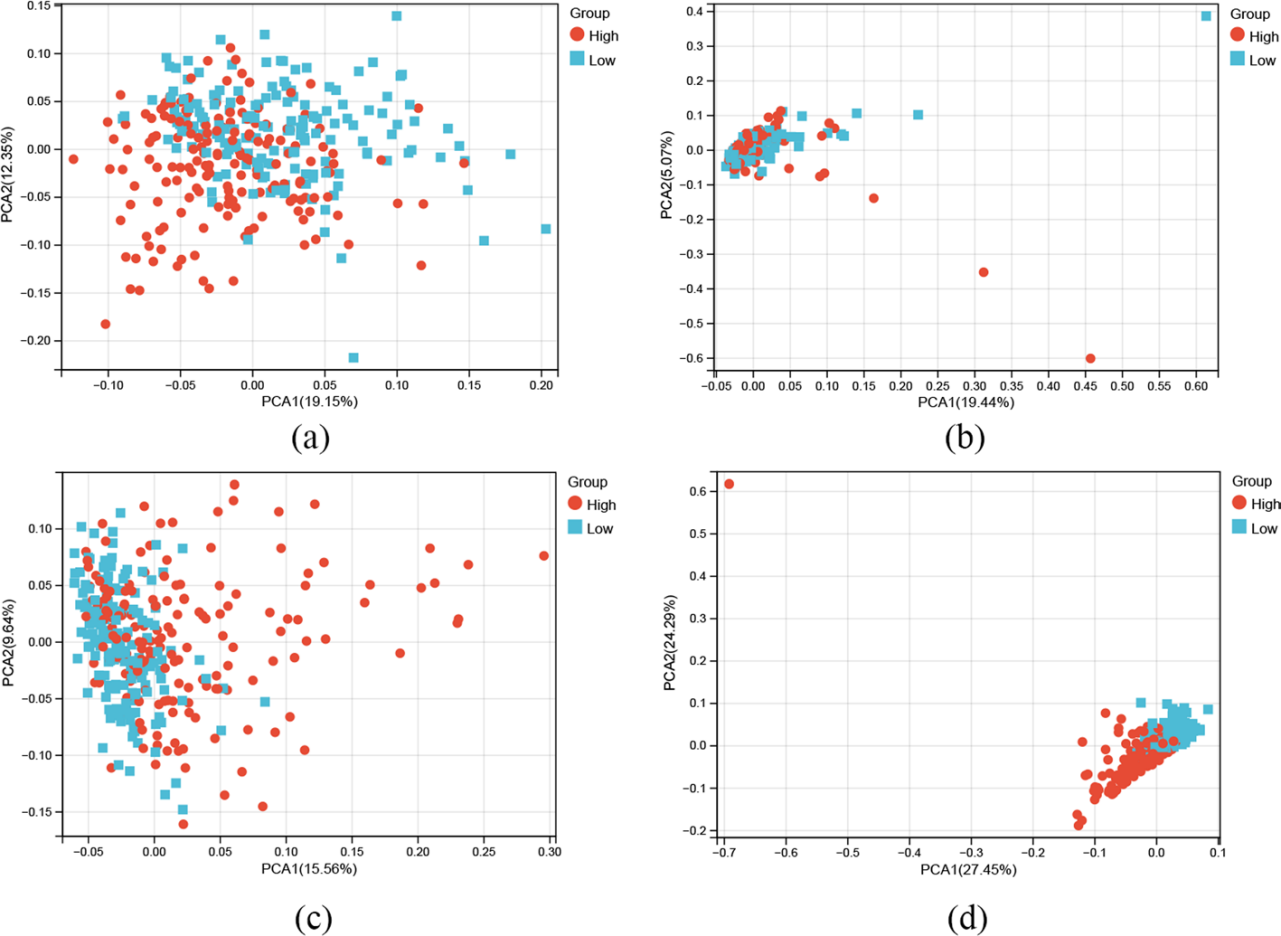
PCA comparison between two groups based on **a** genome-wide, **b** m7G methylation-regulated genes, **c** m7G-related lncRNAs, and **d** m7G-LPS in TCGA entire set

PCA was utilized for the purpose of analyzing the variations between both risk groups in terms of genome-wide expression profiles, m7G methylation-regulated genes expression profiles, prognosis-related m7GlncRNA expression profiles, and seven prognostic m7G-related lncRNAs expression profiles. The findings of this analysis showed clearer differences across the two groups in the seven prognostic m7G-related lncRNAs expression profiles than in the other three expression profiles (Fig. 6). Therefore, seven prognostic m7G-related lncRNAs expression profiles were greatly distinct and could be used to differentiate effectively across the two GC populations.

### Gene set enrichment analysis (GSEA) of m7G-LPS

**Fig. 7:**
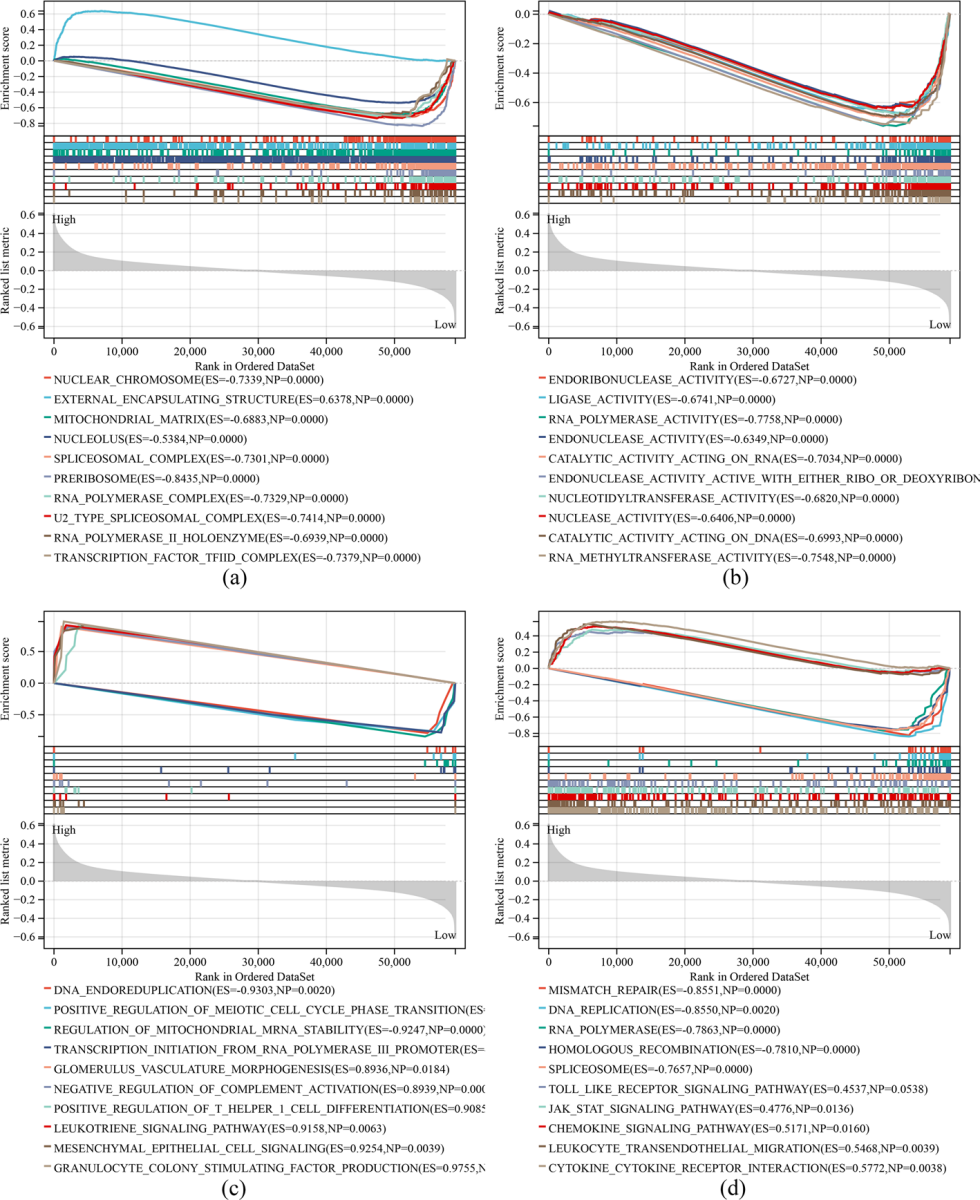
GSEA situation in the two groups. **a** GO: Cellular Component. **b** GO:Molecular Function. **c** GO: Biological Process. **d** KEGG signaling pathways

To clarify the differences in the potential pathways activated in the two risk groups, GSEA was conducted. The top ten signaling pathways (Fig. 7) in the two groups were visualized based on this analysis. Gene Ontology (GO) analysis revealed high enrichment of external encapsulating structure, negative regulation of T cell migration and T helper 1 type immune response, positive regulation of T helper 1 cell differentiation, leukotriene signaling pathway, epithelial-mesenchymal signaling, and granulocyte colony-stimulating factor production in the high-risk group and positive regulation of establishment of protein localization to telomere, DNA endoreduplication, positive regulation of meiotic cell cycle phase transition, regulation of mitochondrial mRNA stability, transcription initiation from RNA polymerase III promoter, endoribonuclease activity, ligase activity, RNA polymerase activity, endonuclease activity, catalytic activity acting on RNA, endonuclease activity active with either RNA or DNA and producing 5 phosphomonoesters, nucleotidyltransferase activity, nuclease activity, catalytic activity acting on DNA, RNA methyltransferase activity, nuclear chromosome, mitochondrial matrix, nucleolus, spliceosomal complex, preribosome, RNA polymerase complex, U2 type spliceosomal complex, small nuclear ribonucleoprotein complex, receptor complex and transcription factor IID complex in the low-risk group. The Kyoto Encyclopedia of Genes and Genomes (KEGG) analysis showed a high enrichment of toll-like receptor signaling pathway, Jak-STAT signaling pathway, chemokine signaling pathway, leukocyte transendothelial migration, cytokine-cytokine receptor interaction in the subjects with a high risk score, while a high enrichment of mismatch repair, DNA replication, RNA polymerase, homologous recombination, and spliceosome in the subjects with a low risk score. These findings indicated the possibility of m7G-LPS’ influence on the course of GC by regulating transcription, translation, and immune infiltration.

### Correlation of the m7G-LPS with clinicopathological features in patients with GC

**Fig. 8:**
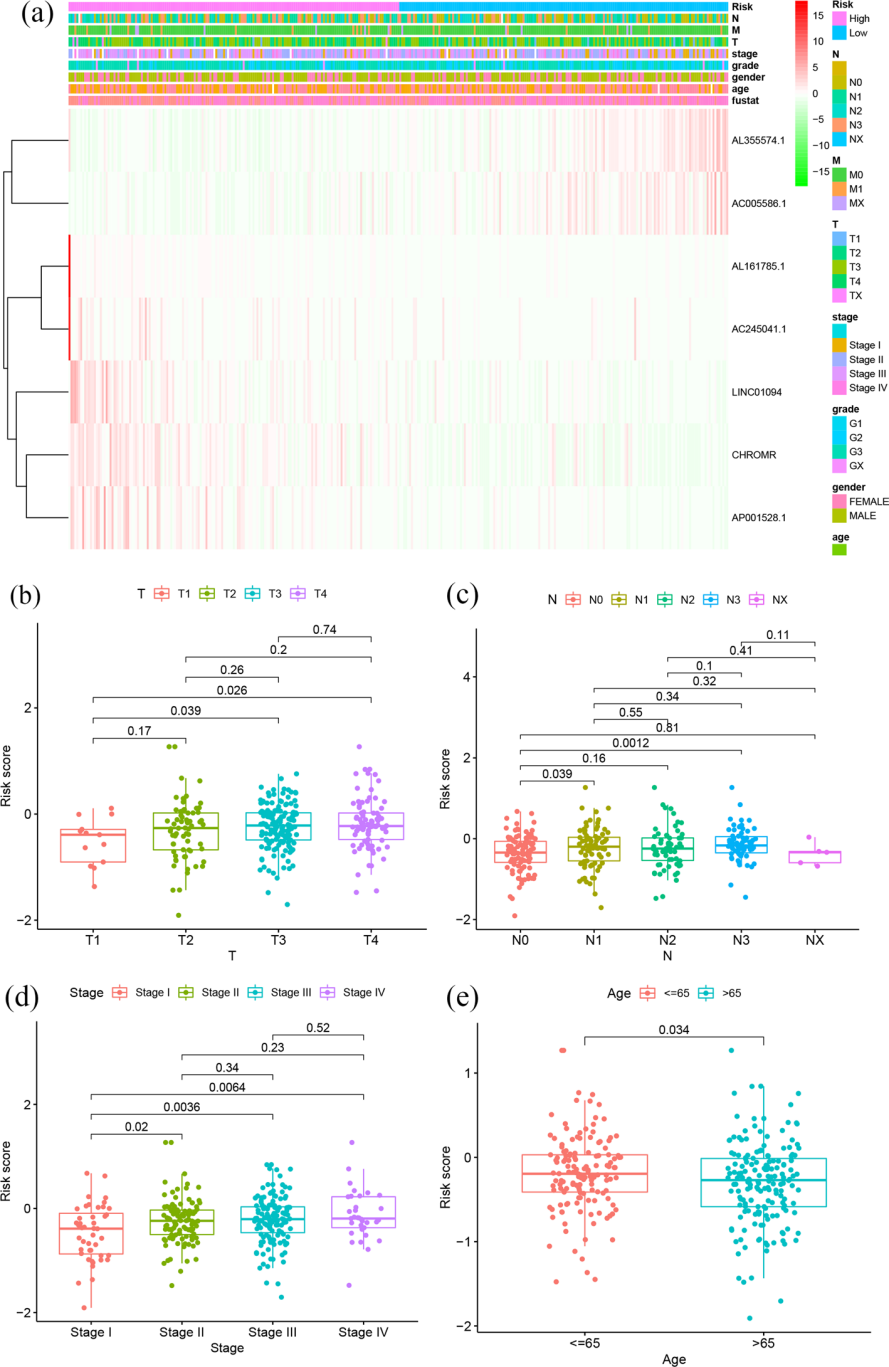
Relationship between risk scores and clinicopathological characteristics. **a** Clinicopathological characteristics and heat map of 7 m7G-LPS expression between the two groups. Relationship between risk score and **b** T-stage, **c** lymph node metastasis, **d** pathological stage and **e** age

The association of the risk score with the clinicopathological features of the two risk groups was investigated. The results suggest a significant difference in T-stage, N-stage, pathological stage, and age between the two groups (Fig. 8a). In particular, GC subjects with T3 and T4 presented an elevated risk score than those with T1 (*p* < 0.05). The subjects with N1 and N3 showed elevated risk scores than those with N0 (*p* < 0.05); and risk scores were remarkably increased in those with STAGE II, STAGE III and STAGE IV than in those with STAGE I (p < 0.05, Fig. 8b, c, d). In addition, patients aged ≤ 65 years presented with a remarkably greater risk score compared to those aged > 65 years group (Fig. 8e). Therefore, high-risk subjects tended to have advanced clinicopathological features.

### Correlation of the m7G-LPS with the immune characteristics of GC patients

**Fig. 9:**
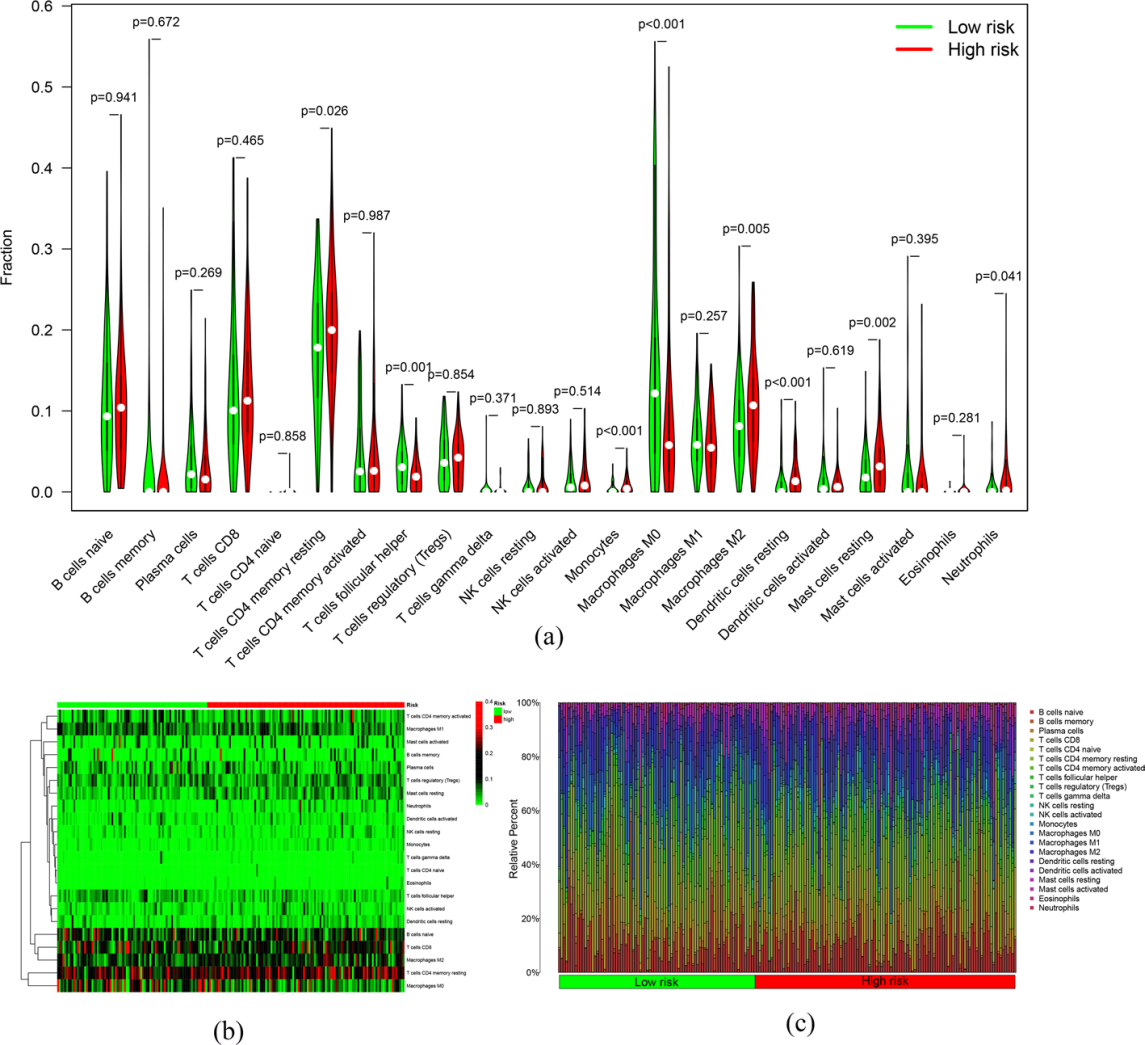
Differences in immune cell infiltration between the two groups. **a** Violin diagram and **b** heat map of immune cell infiltration. **c** Relative percentage of different immune cells between the two groups

**Fig. 10:**
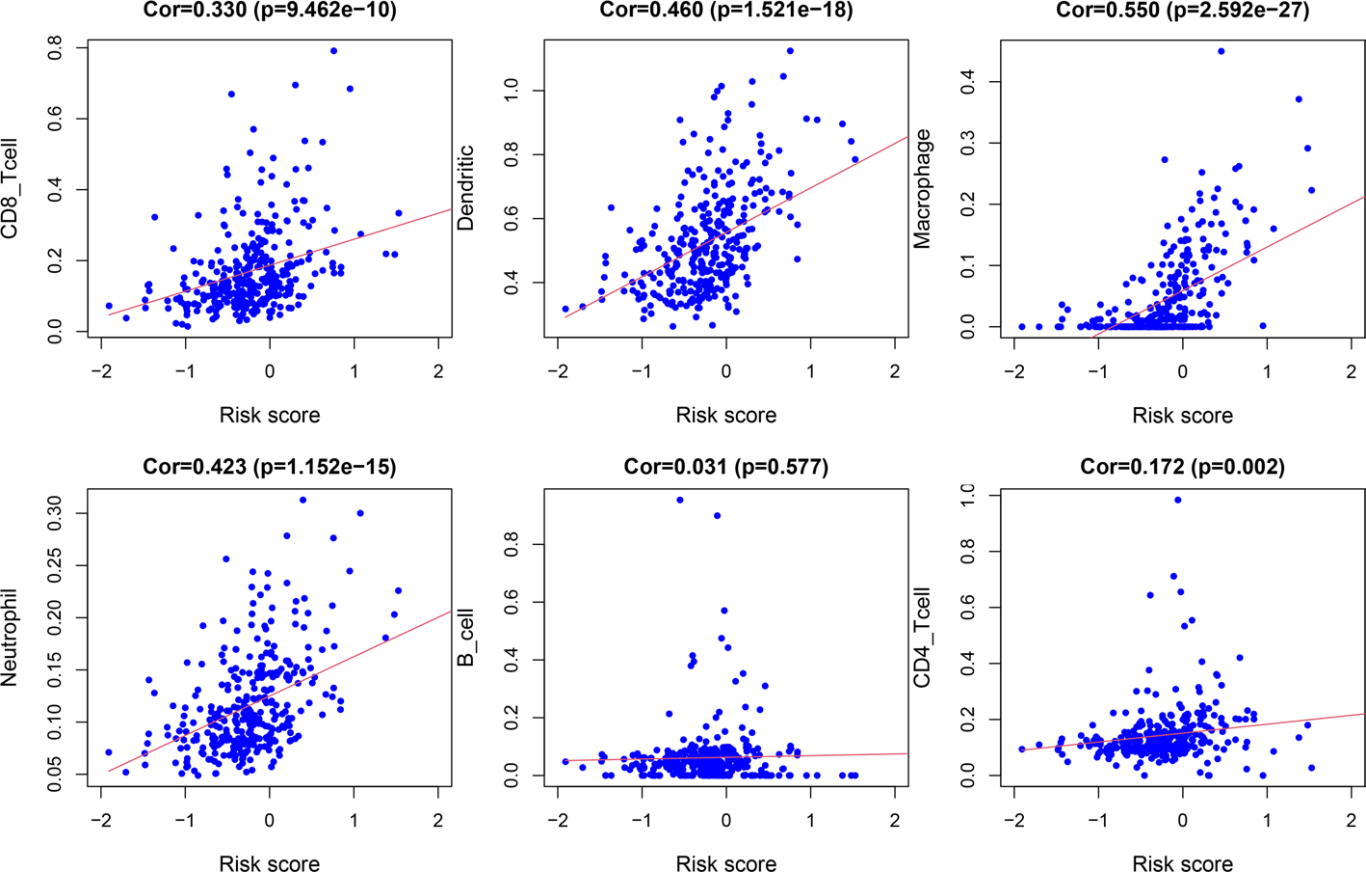
Scatter plot of correlation between risk score and infiltration of CD8 T cells, Dendritic, Macrophage, Neutrophil, B cells and CD4 T cells

To explore the value of the m7G-LPS in the tumor immune microenvironment, the CiberSort algorithm was utilized for analysis of the differences in the distribution of 22 tumor immune cells in the two risk groups. The heat plot and violin plot show that the immune cell distribution differed between them (Fig. 9a, b). The subjects with the high risk presented with higher infiltration abundance of memory CD4 T cells resting, monocytes, M2macrophages, dendritic cells (DCs) resting, mast cells resting, and neutrophils, while low-risk subjects had higher abundance of infiltration M0 macrophages and follicular helper T cells (*p* < 0.05). The immune cell composition of the samples is shown in Fig. 9c. This was followed by an investigation of the association of risk score with immune cells. A positive correlation of risk score was found with the abundance of CD4 T cell (cor = 0.172, *p* < 0.01), CD8 T cell (cor = 0.330, *p* < 0.001), DCs (cor = 0.460, *p* < 0.001), macrophages (cor = 0.550, *p* < 0.001), and neutrophils (cor = 0.423, *p* < 0.001). These findings were indicative of the involvement of immune cells in risk score grading (Fig. 10).

**Fig. 11:**
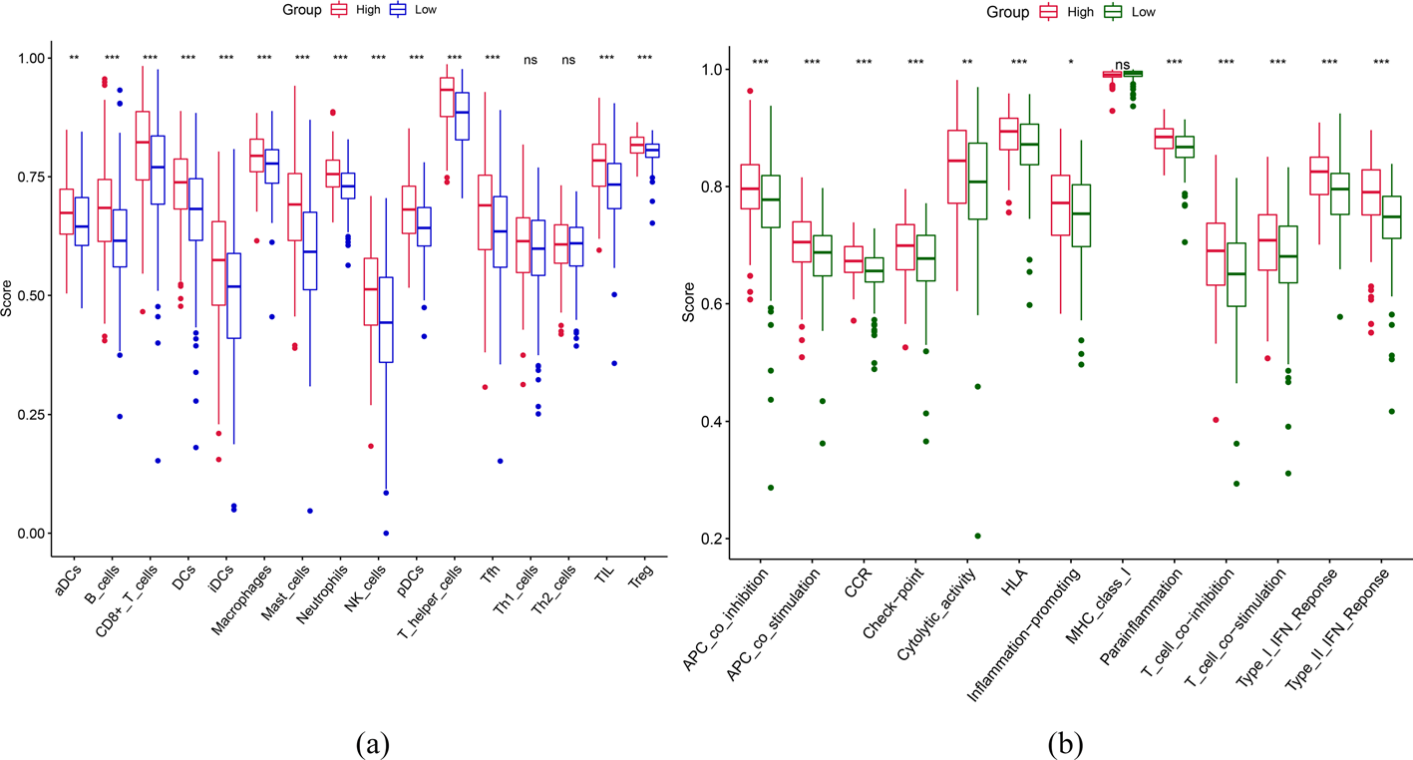
Enrichment scores of **a** immune cells and **b** immune function. (∗ p < 0.05, ∗  ∗ p < 0.01, and ∗  ∗  ∗ p < 0.001)

In addition, the correlation between m7G-LPS and immune cell and immune function concentration scores was also evaluated. The results showed higher enrichment scores of aDCs, B cells, CD8 + T cells, DCs, iDCs, macrophages, mast cells, neutrophils, natural killer (NK) cells, pDCs, T helper cells, TIL, Treg and Tfh in the subjects with high-risk score (p < 0.05, Fig. 11a). Also, greater enrichment scores were observed in the high-risk group for multiple immune functions, such as antigen-presenting cell (APC) co-inhibition, APC co-stimulation, CCR, checkpoint, cytolytic activity, human leukocyte antigen, inflammation-promotion, MHC class I, parainflammation, T cell co-inhibition, T cell co-stimulation, Type I IFN response, and Type II IFN response (p < 0.05, Fig. 11b). These findings were indicative of the involvement of seven prognostic m7G-related lncRNAs in immune function regulation.

### Value of m7G-LPS in immunotherapy and chemotherapy

**Fig. 12:**
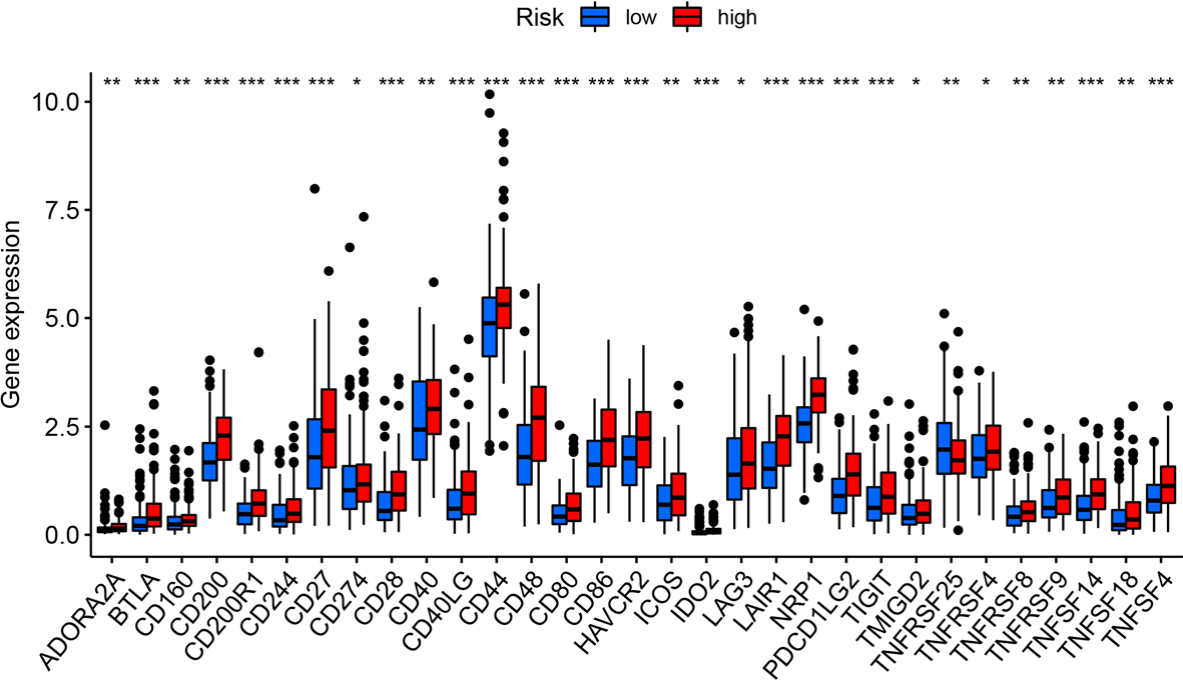
Differences in immune checkpoint gene expression between the two groups. (∗ p < 0.05, ∗  ∗ p < 0.01, and ∗  ∗  ∗ p < 0.001)

**Fig. 13:**
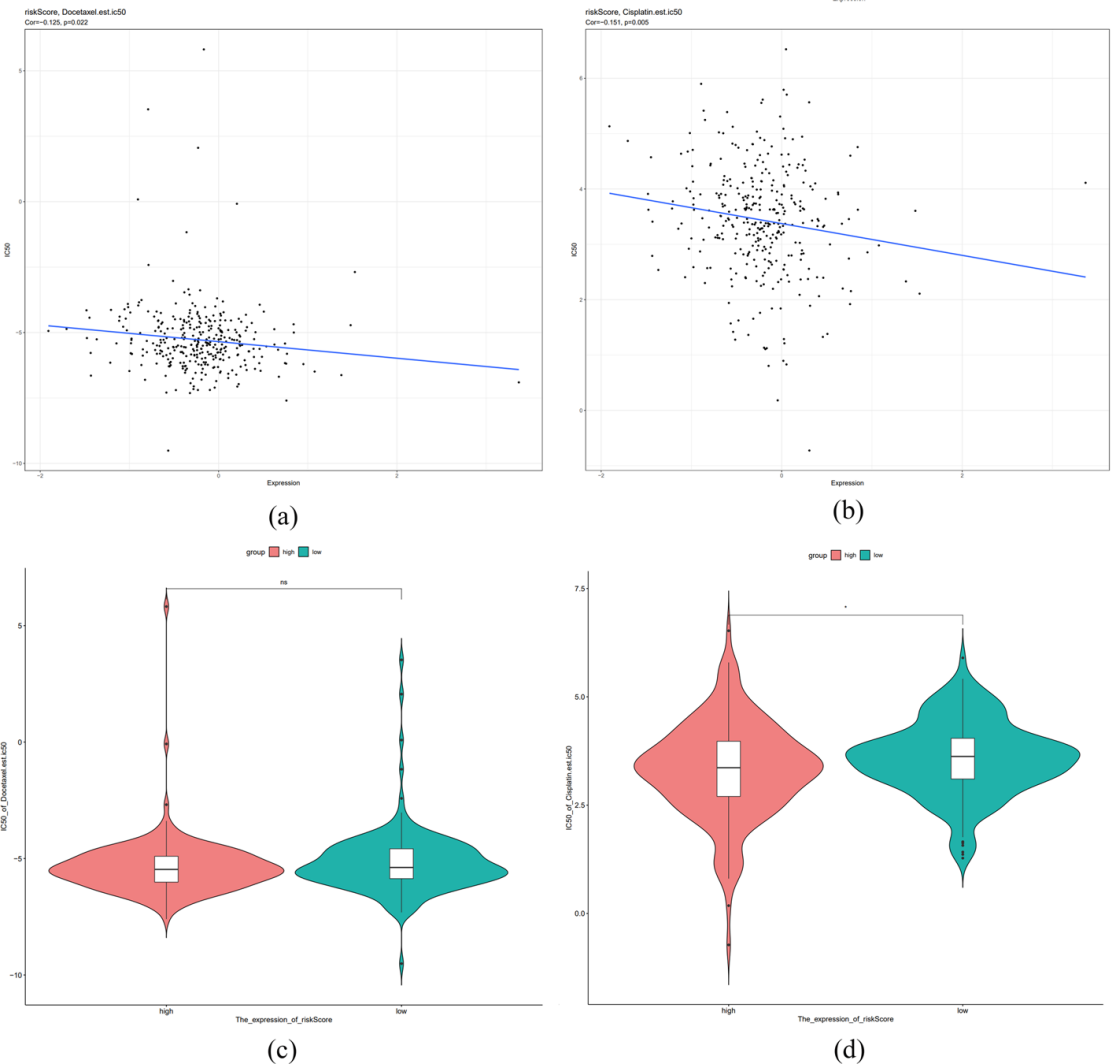
The Value of m7G-LPS in Immunotherapy and Chemotherapy. Scatter plot of correlation between risk scores and IC50 of Docetaxel (**a**) and Cisplatin (**b**). Violin plot of the difference in IC50 of Docetaxel (**c**) and Cisplatin **d** between the two high and low risk groups. (∗ p < 0.05, ∗  ∗ p < 0.01, and ∗  ∗  ∗ p < 0.001)

The value of m7G-LPS in guiding treatment decision-making was also studied. The expression of immune checkpoint genes across the two groups was also assessed. Elevated expression of *ADORA2A, BTLA, CD160, CD200, CD200R1, CD244, CD27, CD274, CD28, CD40, CD40LG, CD44, CD48, CD80, CD86, HAVCR2, ICOS, IDO2, LAG3, LAIR1, NRP1 PDCD1LG2, TIGIT, TMIGD2, TNFRSF4, TNFRSF8, TNFRSF9, TNFSF14, TNFSF18,* and *TNFSF4* was found in the high-risk subjects (*p* < 0.05) in comparison to the low-risk subjects. In contrast, *TNFRSF25* expression was higher in the low-risk subjects (*p* < 0.05). The findings were indicative of the significance of m7G-LPS in predicting the efficiency of immune checkpoint inhibitor therapy (Fig. 12). Moreover, the association of risk score with half maximal inhibitory concentration (IC50) of common chemotherapeutic agents was assessed, and the results revealed a negative association of the IC50 of Cisplatin with the risk score (cor = − 0.125, *p* = 0.022), and it was remarkably decreased in the high-risk compared to the other group (*p* < 0.05, Fig. 13b, d). However, although the IC50 of Docetaxel was also negatively correlated with risks core (cor = − 0.151, p < 0.05), no remarkable variations appeared in the IC50 across the two groups (Fig. 13a, c). These findings are suggestive of the increased chemotherapy sensitivity of low-risk patients as well as better prognoses and clinical outcomes.

### Expression of m7G-related lncRNAs in tissues

**Fig. 14:**
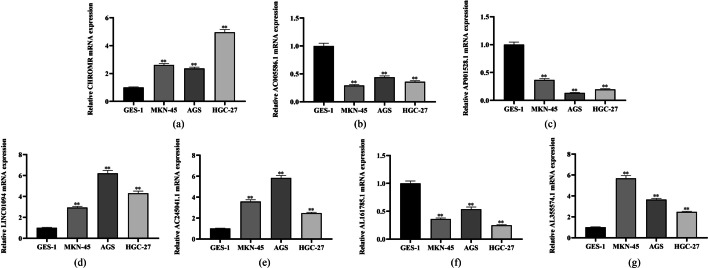
qRT–PCR of **a** CHROMR, **b** AC005586.1, **c** AP001528.1, **d** LINC01094, **e** AC245041.1, **f** AL161785.1, **g** AL355574.1 in GSE-1, MKN-45, AGS and HGC-27 cell lines. (∗ p < 0.05, ∗  ∗ p < 0.01, and ∗  ∗  ∗ p < 0.001)

The expression of m7G-related lncRNAs was assessed in four cell lines, namely, GSE-1, MKN-45, AGS, and HGC-27, by q-PCR. Their expression varied remarkably between the cancerous and healthy cell lines (Fig. 14). Among them, CHROMR, LNC01094, AC245041.1, and AL355574.1 had significantly higher expression levels in tumor cell lines, whereas AC005586.1, AL16178.5, and AP001528.1 had the opposite, which is consistent with the results of our analysis. This result further validated the accuracy of the developed risk model.

## Discussion

Gastric cancer is among the top five malignant tumors in terms of morbidity and mortality worldwide [31]. A lot of research has been conducted on the early diagnosis, treatment, and prognosis evaluation of GC, but the molecular mechanism of GC development remains unclear [32]. A large number of researches confirmed lncRNAs’ importance in GC onset and advancement. Liu et al. demonstrated that the lncRNA HOTAIR leads to epigenetic inactivation of miR-34a, causing activation of epithelial–mesenchymal transition (EMT) in cancer cells using the HGF/c-MET/SNAIL pathway [33]. The increased expression of MALAT1 in GC cells reduces the inhibitory impact of UPF1 on cell proliferation and EMT and increases apoptosis, resulting in GC cell invasion and metastasis [34]. LncRNAs have high specificity and sensitivity; therefore, they have the potential to serve as biomarkers for early screening, diagnosis, treatment, prognosis, and drug response to various diseases. Tan et al. showed a remarkable association of lncRNA GACAT2 expression with lymph node and distant metastasis, as well as neuroinvasion in GC [35]. Ji et al. demonstrated a significant association of LINC00086 expression levels with tumor size, lymph node metastasis, TNM stage, and CEA and CA19-9 levels [36]. However, many lncRNAs are still to be discovered as prognostic markers for GC.

Methylation affects almost all aspects of RNA processing and is essential for regulating gene expression, maintaining RNA stability, and homeostasis in vivo. Increasing evidence shows the association of lncRNA’s abnormal expression with tumorigenesis. YAN et al. showed that METTL14 knockout abolished the m6A level of lncRNA XIST and enhanced the expression of lncRNA XIST, leading to colorectal cancer proliferation and metastasis [37]. Hang et al. showed that m6A RNA methylation maintains RMRP stability through the TGFBR1/SMAD2/SMAD3 pathway, which ultimately leads to the advancement and progression of non-small cell lung cancer [38]. As an important RNA modification, m7G methylation has been shown to be associated with many cellular processes that lead to cancer progression. METTL1 mediates m7G methylation in miRNAs and promotes tumor cell migration [8]. However, the mechanism of the pathogenicity of m7 G and lncRNAs in GC onset and progression is still unclear. Our study therefore focuses on the ability of m7G-related lncRNAs as gastric cancer biomarkers to better understand the role of m7G methylation in gastric cancer and thus provide a possible basis for further therapeutic interventions. During this research, the GC patients were stratified into different subgroups based on m7G-related lncRNA expression, a prognostic model was constructed, and its utility for guiding GC diagnosis and treatment was checked.

The GC transcript data were obtained from TCGA, 22 m7G methylation-regulated genes were identified based on published literature, and the differences in expression in GC and healthy subjects were analyzed. Univariate and multivariate COX regression analyses helped in identifying 30 predictive m7G-associated lncRNAs. Lasso regression analysis helped build a risk prediction model on the basis of seven shortlisted m7G-associated lncRNAs (AL161785.1, LINC01094, CHROMR, AP001528.1, AC245041.1, AL355574.1, AC005586.1) to obtain risk scores for GC patients and a nomogram was constructed based on COX regression. According to time-dependent ROC, clinical ROC, and the calibration plot, the constructed nomogram had a reliable predictive ability. Kaplan–Meier curves, independent prognostic analysis, PCA, and q-PCR results further confirmed the reliability of the established m7G-LPS as a prognostic marker. Next, the GC subjects were stratified into two groups of high and low-risk, and their differences in clinicopathologic features were analyzed. The high-risk patients presented with a greater likelihood of developing a more advanced pathologic stage. These findings were suggestive of the impact of lncRNAs in the m7G-LPS on the progression and prognosis of GC and their possible therapeutic importance.

Of the lncRNAs used to establish the m7G-LPS in this study, AL161785.1, LINC01094, CHROMR, AP001528.1, AC245041.1 were risk genes, while AL355574.1, AC005586.1 were considered as protective genes. LINC01094 was found to play a role as a cancer-promoting factor in many tumors. Xia et al. revealed that LINC01094 directly targets miR-340-5p and negatively regulates its expression, promoting breast cancer cell proliferation and cell cycle advancement and suppressing apoptosis [39]. In ovarian cancer, the LINC01094/miR-577 axis regulates the expression of a β-linked protein, c-Myc, and cell cycle protein D1, promoting cancer cell proliferation, invasion, and migration [40]. Yufeng et al. demonstrated that LINC01094 acts as a competitive endogenous RNA in clear cell renal cell carcinoma and plays a tumor-promoting role through the competitive link to miR-224-5p for the regulation of CHSY1 expression [41]. Moreover, it was found that some of m7G-LPS related lncRNAs are involved in constituting other GC prognostic models. LINC01093 and CHROMR, as necroptosis-related lncRNAs, constitute a predictive model with ten other lncRNAs [42]. AC245041.1 could potentially be associated with tumor angiogenesis, suggesting a poor prognosis for GC [7]. AL355574.1, as a ferroptosis- and cellular senescence-related lncRNA, constitute a predictive model for GC [43, 44]. However, the role of AL161785.1, AP001528.1, and AC005586.1 in tumors remains to be confirmed, and the findings of this study direct toward a new reference to conduct future research.

During this research, GSEA findings were indicative of the involvement of m7G-LPS in the regulation of several pathways, such as the toll-like receptor signaling pathway, JAK-STAT signaling pathway, chemokine signaling pathway, leukocyte transendothelial migration, granulocyte colony-stimulating factor production, to influence the course of GC through multiple immune pathways. Immune cell infiltration analysis revealed remarkably increased infiltration of memory CD4 T cells resting, monocytes, M2macrophages, DCs resting, mast cells resting, and neutrophils in high-risk patients compared to others showing their positive correlation with the risk score. M2 macrophages produce anti-inflammatory cytokines so as to inhibit immune surveillance of tumor cells and promote angiogenesis and stromal remodeling, facilitating tumor progression and metastasis [45]. CD4 resting memory T cells, resting mast cells, and resting DCs may also contribute to tumor progression to the progressive stage. This result suggests that several immune cells may be involved in the progression of GC. However, the enrichment of M1 macrophages and follicular helper T cells was greater in the low-risk subjects. M1-type macrophages manifest anti-tumor effects through the secretion of pro-inflammatory cytokines and chemokines and the presentation of antigens exclusively for the purpose of participating in a positive immune response and mediating immune surveillance [46]. Follicular helper T cells play an anti-tumor role by promoting B-cell differentiation and inducing humoral immunity [47]. This may be the cause behind the improved prognosis of low-risk subjects in comparison to the others. m7G-LPS impact on immune cells and immune function scores was also studied. Many immune cells, such as mast cells, iDC, NK cells, and follicular helper T cells, had greater enrichment scores in the subjects with a high risk. Some immune function scores, such as CCR, inflammation promotion, and T cell co-inhibition, were also remarkably increased in high-risk populations. These results suggest that m7G-LPS is involved in regulating many immune cells and immune functions. This might also explain why subjects with varying risk scores respond differentially to immunotherapy.

LncRNAs have been shown to be vital for immune recognition and the escape of tumor cells from the immune system [48]. Moreover, remarkable variations in the expression of 31 immune checkpoint genes were observed across the two risk groups. Therefore, m7G-LPS may provide a reference in the prediction of immune checkpoint inhibitor treatment efficiency in GC patients. A negative association of the risk scores was observed with the IC50 of both cisplatin and docetaxel, and subjects with a high risk score appeared to be less sensitive to chemotherapeutic agents. This study suggests the possible predictive capability of m7G-LPS for immunotherapy and chemotherapy.

With the increasing studies on the mechanism of gastric carcinogenesis, ceRNAs have been shown to play an important role in various aspects of gastric carcinogenesis and invasion and metastasis. However, the regulation of non-coding RNAs is not isolated, but multiple factors are interrelated and work together, and this complex regulatory relationship poses many difficulties for experimental validation. With the development of bioinformatics analysis tools such as machine learning, deep learning and convolutional neural networks, bioinformatics analysis of lncRNA–miRNA will bring great reference value to experiments. Several methods have been proposed for predicting lncRNA–miRNA interactions, such as lncRNA–miRNA interactions prediction by logistic matrix factorization with neighborhood regularized (LMFNRLMI), graph convolutional neural network and conditional random field (GCNCRA), and network distance analysis model for lncRNA–miRNA association prediction (NDALMA), all of which have been shown to be reliable [49–51]. In addition, several bioinformatics tools provide an important contribution to tumor metabolism analysis and drug development. A bioinformatics tool, named graph convolutional network with graph attention network (GCNAT), is able to predict hERG channel blockers in the early stages of drug discovery [52]. The metabolite-disease associations predicted by the graph convolutional network with graph attention network (GCNAT) method have also been experimentally validated [53].

This study is limited because the constructed model lacked validation on non-TCGA datasets, mainly due to the lack of datasets with complete lncRNA sequencing data. Second, there is a need to conduct more molecular biology experiments and clinical trials to further validate the findings of this study.

## Conclusion

During this research, seven prognostic m7G-related lncRNAs with a high correlation with the prognosis of GC patients based on the TCGA database and the role of m7G-LPS in the prediction of survival rate, correlation with tumor immune microenvironment, possible underlying mechanisms of m7G-related lncRNAs, prediction of potential immunotherapy targets, and sensitivity of chemotherapeutic drugs were studied. It can be postulated that the m7G-LPS established in this study can serve as a predictor of the survival rate of GC patients and may facilitate future individualized treatment.

## Data Availability

This study analyzed data from The Cancer Genome Atlas (TCGA) (https://portal.gdc.cancer.gov/). These data are free and publicly available.
